# Supplementary material for: Structural basis of Gabija anti-phage defence and viral immune evasion
Source: Nature. 2023 Nov 22;625(7994):360–5. doi: 10.1038/s41586-023-06855-2 (PMC10781630; doi:10.1038/s41586-023-06855-2)

nature portfolio

https://doi.org/10.1038/s41586-023-06855-2

Supplementary information

Structural basis of Gabija anti-phage defence and viral immune evasion

In the format provided by the authors and unedited

a

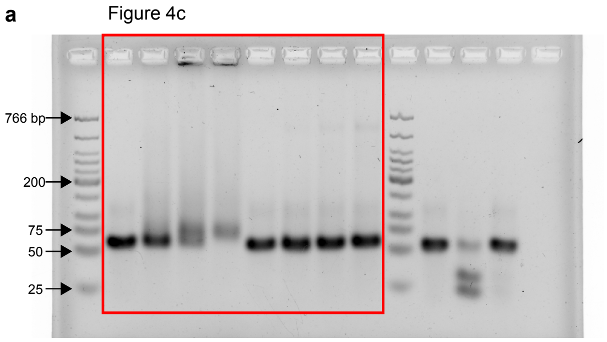

Supplement: Supplementary file 1 — Uncropped gels. [file 41586_2023_6855_MOESM1_ESM.pdf]
